# Efficacy and Safety of Inclisiran in Patients with Polyvascular Disease: Pooled, Post Hoc Analysis of the ORION-9, ORION-10, and ORION-11 Phase 3 Randomized Controlled Trials

**DOI:** 10.1007/s10557-022-07413-0

**Published:** 2022-12-23

**Authors:** Wolfgang Koenig, Lorena Garcia Conde, Ulf Landmesser, Lawrence A. Leiter, Kausik K. Ray, Gregory G. Schwartz, R Scott Wright, Jackie Han, Frederick J. Raal

**Affiliations:** 1 Deutsches Herzzentrum München, Technische Universität München, DZHK (German Centre for Cardiovascular Research), Partner Site Munich Heart Alliance, Munich, Germany; 2 Institute of Epidemiology and Medical Biometry, University of Ulm, Ulm, Germany; 3 Novartis Pharma AG, Basel, Switzerland; 4 Department of Cardiology, Charité-University Medicine Berlin, Berlin Institute of Health (BIH), DZHK, Partner Site, Berlin, Germany; 5 Li Ka Shing Knowledge Institute, St. Michael’s Hospital, University of Toronto, Toronto, Canada; 6 Imperial Centre for Cardiovascular Disease Prevention, Department of Primary Care and Public Health, Imperial College, London, UK; 7 Division of Cardiology, University of Colorado School of Medicine, Aurora, CO USA; 8 Division of Preventive Cardiology and Department of Cardiology, Mayo Clinic, MN Rochester, USA; 9 Novartis Pharmaceuticals Corp, East Hanover, NJ USA; 10 Faculty of Health Sciences, University of the Witwatersrand, Johannesburg, South Africa

**Keywords:** Inclisiran, LDL-C, ASCVD, Polyvascular disease

## Abstract

**Purpose:**

Patients with polyvascular disease (PVD) are at very high cardiovascular risk and require intensive lipid-lowering therapy. This analysis describes the lipid-lowering efficacy and safety of inclisiran versus placebo in patients with and without PVD.

**Methods:**

In this post hoc analysis of the ORION-9, ORION-10, and ORION-11 trials, patients were randomized 1:1 to receive 284 mg inclisiran (300 mg inclisiran sodium) or placebo on day 1, day 90, and 6-monthly thereafter. Percentage change in low-density lipoprotein cholesterol (LDL-C) from baseline to day 510 and corresponding time-adjusted change from day 90 and up to day 540 were evaluated per patients’ PVD status. Safety was assessed over 540 days.

**Results:**

Of 3454 patients, 470 (13.6%) had PVD, and 2984 (86.4%) did not. Baseline characteristics were generally balanced between the treatment arms in both cohorts. A greater proportion of patients with PVD had comorbidities versus those without. The mean (95% confidence interval [CI]) placebo-corrected LDL-C percentage change from baseline to day 510 was −48.9% (−55.6 to −42.2) in patients with PVD and −51.5% (−53.9 to −49.1) in patients without. Proportions of patients with reported treatment-emergent adverse events (TEAEs) and treatment-emergent serious adverse events were similar between treatment arms, irrespective of PVD status, except for an excess of mild or moderate clinically relevant TEAEs at the injection site with inclisiran.

**Conclusion:**

Twice-yearly inclisiran dosing (after the initial and 3-month doses) was well tolerated and provided effective and sustained lipid-lowering in patients, irrespective of PVD status.

**Supplementary Information:**

The online version contains supplementary material available at 10.1007/s10557-022-07413-0.

## Introduction

Atherosclerotic cardiovascular disease (ASCVD) is the leading cause of mortality and morbidity worldwide, exerting a substantial healthcare and economic burden [1, 2]. Patients with ASCVD, particularly those with more severe disease, experience increased mortality and have a reduced quality of life [3–5]. The presence of atherosclerotic plaques in at least two major artery beds (coronary, peripheral, or cerebrovascular) is defined as polyvascular disease (PVD), which affects ~15–30% of patients with ASCVD [6–8].

PVD is an independent predictor of an increased risk of cardiovascular (CV) events [6–8], stronger than diabetes or prior ischemic events [8–10]. The presence of PVD was associated with a 99% increased risk of CV death, myocardial infarction (MI), and stroke in the global Reduction of Atherothrombosis for Continued Health (REACH) Registry analysis, while diabetes and prior ischemic events were associated with a 44% and 71% greater risk, respectively, at 4-year follow-up [8, 10]. Recent analyses from the MarketScan and Medicare databases showed that the incidence of CV events increases with the number of affected vascular beds, with an ASCVD event rate per 1000 person-years of 40.8 (95% confidence interval [CI], 33.2–36.2), 68.9 (95% CI, 67.9–70.0), and 119.5 (95% CI, 117.0–122.0) for 1, 2, or 3 affected beds, respectively [9, 11].

Despite the availability of a number of guideline-recommended therapies for patients with clinical ASCVD or increased CV risk [12, 13], a substantial residual risk of CV events remains, which is the highest in patients with PVD [14, 15]. This population also tends to have higher lipoprotein(a) [Lp(a) levels [16], and thus, a more aggressive lipid-lowering therapeutic approach is justified from a secondary prevention standpoint. Due to its causal effect, guidelines strongly recommend lowering low-density lipoprotein cholesterol (LDL-C) levels to reduce the risk of both first and recurrent CV events in patients with ASCVD [12, 13, 17, 18]. Statins are recommended as first-line therapy, followed by the addition of ezetimibe if guideline-recommended LDL-C levels are not achieved with the maximally tolerated dose (MTD) of statins [13]. However, despite available lipid-lowering therapy (LLT), a substantial proportion of patients with clinical ASCVD do not achieve guideline-recommended LDL-C goals [19]. Analysis from the DA VINCI registry showed that only 22% of patients with very high CV risk treated with high-intensity statins achieved recommended LDL-C levels [19]. A major factor impacting LDL-C lowering in clinical practice relates to long-term non-adherence to LLT [20]. Developing therapies with dosing regimens that do not significantly contribute to the medication burden for patients could be an advantageous treatment strategy to achieve long-term LDL-C goals [21].

New therapies targeting proprotein convertase subtilisin/kexin type 9 (PCSK9) have been previously shown effective in lowering LDL-C levels and CV events [22, 23]. Inclisiran is a first-in-class small-interfering ribonucleic acid (siRNA) agent that targets PCSK9 hepatic messenger RNA and prevents the production of PCSK9 protein, resulting in an increased surface concentration of LDL receptors and hepatic uptake of LDL-C [24]. Inclisiran provides effective and sustained reduction in LDL-C levels with twice-yearly dosing (after the initial and 3-month doses) and is well tolerated in patients with heterozygous familial hypercholesterolemia (HeFH), ASCVD, and ASCVD risk equivalent [25–28]; whether inclisiran reduces CV events is under investigation in dedicated CV outcome trials.

Recent studies have demonstrated the substantial impact of therapies targeting PCSK9 in patients with PVD [16, 29], and it is critical to understand if the LDL-C-lowering efficacy of inclisiran is also preserved in these patients. A post hoc analysis of pooled data from three phase 3 studies (ORION-9 [NCT03397121], ORION-10 [NCT03399370], and ORION-11 [NCT03400800]) is presented here, describing the efficacy and safety of inclisiran versus placebo in subpopulations of patients with and without PVD.

## Methods

### Study Design

This was a post hoc analysis of pooled data from the ORION-9, ORION-10, and ORION-11 trials. These trials had matching study designs and assessment schedules (Supplementary Fig. 1), which have been previously described [25–27]. Briefly, the three phase 3 studies were randomized, double-blinded, placebo-controlled trials, with a duration of 18 months to evaluate the efficacy and safety of inclisiran.

All participants were randomized 1:1 to receive 284 mg inclisiran (equivalent to 300 mg of inclisiran sodium) or placebo, in combination with background statins with or without other LLT, such as ezetimibe. Study treatments were administered subcutaneously on day 1, day 90, and 6-monthly thereafter. Additional clinic visits were conducted on days 30, 150, 330, 450, 510, and 540 for follow-up and laboratory assessments.

For this post hoc analysis, patients were stratified based on their PVD status, where PVD was defined as the presence of ≥2 of the following conditions: peripheral artery disease (PAD), coronary artery disease (CAD), or cerebrovascular disease (CeVD; definitions are described in Supplementary Table 1). The No PVD cohort included patients with only PAD, only CeVD, only CAD, or ASCVD risk equivalent. Patients with missing information on one of the three categories (PAD, CAD, or CeVD) in the medical history section of the case report form (CRF) were excluded from this analysis.

### Population

Detailed inclusion and exclusion criteria have been previously described [25]. Briefly, participants were required to be ≥18 years with a history of HeFH, ASCVD, or ASCVD risk equivalent (defined as type 2 diabetes mellitus, familial hypercholesterolemia, or >20% 10-year risk of a CV event, as assessed using the Framingham CV disease risk score or equivalent) and elevated LDL-C levels (≥100 mg/dL [≥2.6 mmol/L] for HeFH and ASCVD risk equivalent; ≥70 mg/dL [≥1.8 mmol/L] for ASCVD) at screening, despite MTD of statins. The MTD was defined as the maximum dose of statin that could be taken regularly without the occurrence of intolerable treatment-emergent adverse events (TEAEs). For patients not on the MTD of statins, documentation of statin intolerance was required, defined as intolerance to all doses of at least two different statins. Treatment with anti-PCSK9 monoclonal antibodies within 90 days of screening was exclusionary.

### Endpoints

The pre-specified co-primary endpoints were the percentage change in LDL-C from baseline to day 510 and the time-adjusted percentage change in LDL-C from baseline after day 90 and up to day 540. The time-adjusted percentage change was calculated as the average of measurements from baseline over the period after day 90 and up to day 540 (days 150, 270, 330, 450, 510, and 540) to assess if the lipid-lowering efficacy of inclisiran is sustained long term over a 6-month period with a twice-yearly dosing regimen. Key secondary endpoints included the absolute change in LDL-C from baseline to day 510, time-adjusted absolute change in LDL-C from baseline after day 90 and up to day 540, and percentage changes in PCSK9, total cholesterol, apolipoprotein B (apoB), and non-high-density lipoprotein cholesterol (non-HDL-C) levels from baseline to day 510. Other secondary endpoints included the percentage change in very-low-density lipoprotein cholesterol (VLDL-C) and triglyceride levels from baseline to day 510 and Lp(a) levels from baseline to day 540; the proportion of patients achieving pre-specified LDL-C levels (<25 mg/dL, <50 mg/dL, <70 mg/dL, and <100 mg/dL); and the proportion of patients achieving ≥50% reduction in LDL-C levels from baseline.

Safety analyses included the proportion of patients with TEAEs, treatment-emergent serious adverse events (TESAEs), and clinically relevant laboratory measurements. Reported TEAEs were defined using the Medical Dictionary for Regulatory Activities standardized terms by system organ classification.

### Statistical Analysis

Baseline characteristics and efficacy were evaluated in the intention-to-treat population, which comprised all randomized patients. Missing data were infrequent but imputed for the analysis of co-primary endpoints. Safety was analyzed in the safety population, which comprised all patients who received ≥1 dose of the study drug. The first co-primary endpoint (percentage change in LDL-C from baseline to day 510) was analyzed using an analysis of covariance (ANCOVA) including treatment, study, and baseline value as a covariate in the model with a multiple imputation washout model for missing data. The second co-primary endpoint (time-adjusted percentage change in LDL-C from baseline after day 90 and up to day 540) was analyzed using mixed models for repeated measures (MMRM) including treatment, visit, treatment-by-visit interaction, study, and baseline value as covariates and a control-based pattern mixture model (CB-PMM) for missing data imputation. An unstructured covariance matrix was utilized. The key secondary endpoint, absolute change in LDL-C from baseline to day 510, was analyzed using an ANCOVA model with a multiple imputation washout model for missing data. An MMRM model that assumes that missing data are missing at random was used for other secondary endpoints including percentage changes in PCSK9, total cholesterol, triglycerides, apoB, non-HDL-C, and VLDL-C. Analysis of percentage changes in Lp(a) was performed using a quantile regression model based on observed data without imputation. The proportions of patients with ≥50% reduction in LDL-C were analyzed using a logistic regression model; the proportions of individuals attaining pre-specified LDL-C levels were summarized and reported as a percentage. A two-sided significance level of alpha = 0.05 was used. Analyses were performed using SAS version 9.4.

## Results

### Patients

This post hoc pooled analysis comprised data from 3454 patients from the three phase 3 trials, of whom 470 (13.6%) had PVD and 2984 (86.4%) did not. Baseline demographic and clinical characteristics were generally balanced between the treatment arms for both cohorts (Table 1). In the PVD cohort, lipid measures were balanced between treatment arms. Median interquartile range (IQR) Lp(a) levels were numerically higher in the inclisiran arm (93.0 [24 to 20]) compared with placebo (51.5 [19 to 194]), but this was not significant (*p* = 0.25; Table 1). A numerically greater proportion of patients with PVD had ASCVD manifestations and CV risk factors than those without, in particular PAD, CeVD, and ischemic stroke (Table 1). Despite more CV risk factors, the use of LLTs was similar in those with and without PVD (Table 1). Baseline LDL-C levels were numerically lower in patients with PVD (102.2–104.0 mg/dL) compared with those without (110.1–111.2 mg/dL); this was not statistically significant (*p* = 0.65; Table 1).

**Table 1:** Baseline demographic and clinical characteristics

Characteristic	PVD		No PVD	
	Inclisiran (*n* = 228)	Placebo (*n* = 242)	Inclisiran (*n* = 1506)	Placebo (*n* = 1478)
Age, years, mean ± SD	67.4 ± 8.5	66.9 ± 8.7	63.7 ± 10.1	63.4 ± 10.0
Male, *n* (%)	152 (66.7)	168 (69.4)	1029 (68.3)	1025 (69.4)
BMI, kg/m^2^, mean ± SD	30.3 ± 6.1	30.4 ± 5.6	30.4 ± 5.6	30.7 ± 5.8, *n* = 1476
eGFR, mL/min/1.73 m^2^, median (IQR)	73.5 (57 to 87)	76.0 (61 to 87)	78.0 (67 to 92)	78.0 (66 to 91)
ASCVD manifestations and CV risk factors, *n* (%)				
ASCVD	228 (100.0)	242 (100.0)	1323 (87.8)	1311 (88.7)
PAD	110 (48.2)	115 (47.5)	58 (3.9)	42 (2.8)
CeVD	157 (68.9)	152 (62.8)	110 (7.3)	92 (6.2)
CHD	221 (96.9)	235 (97.1)	1148 (76.2)	1175 (79.5)
Myocardial infarction	125 (54.8)	133 (55.0)	706 (46.9)	729 (49.3)
Ischemic stroke	117 (51.3)	112 (46.3)	99 (6.6)	78 (5.3)
Hypertension	206 (90.4)	223 (92.1)	1182 (78.5)	1159 (78.4)
Heart failure	51 (22.4)	51 (21.1)	151 (10.0)	173 (11.7)
Diabetes mellitus	118 (51.8)	106 (43.8)	503 (33.4)	459 (31.1)
Current smoker	51 (22.4)	52 (21.5)	241 (16.0)	203 (13.7)
Lipid-lowering *n* (%)				
Statin use	211 (92.5)	222 (91.7)	1395 (92.6)	1362 (92.2)
High-intensity statin use	167 (73.2)	184 (76.0)	1128 (74.9)	1094 (74.0)
Ezetimibe use	22 (9.6)	33 (13.6)	225 (14.9)	229 (15.5)
Laboratory measurements, mean ± SD				
LDL-C, mg/dL	102.2 ± 44.8	104.0 ± 38.1	111.2 ± 42.0	110.1 ± 43.5
Total cholesterol, mg/dL	178.0 ± 50.2	181.6 ± 44.9, *n* = 241	189.1 ± 47.7, *n* = 1502	187.3 ± 48.5, *n* = 1475
Non-HDL-C, mg/dL	132.3 ± 49.2	134.1 ± 45.4, *n* = 241	140.2 ± 46.4, *n* = 1502	139.4 ± 47.4, *n* = 1475
HDL-C, mg/dL	45.7 ± 13.9	47.4 ± 15.4, *n* = 241	48.9 ± 15.0, *n* = 1502	47.9 ± 13.8, *n* = 1475
VLDL-C, mg/dL	28.7 ± 15.7	29.5 ± 17.1	28.0 ± 16.0, *n* = 1500	28.3 ± 15.4, *n* = 1471
ApoB, mg/dL	93.4 ± 28.3, *n* = 227	95.2 ± 26.6	98.7 ± 27.9, *n* = 1502	98.0 ± 28.1, *n* = 1476
Triglycerides, mg/dL	151.1 ± 77.6	149.4 ± 79.8	145.6 ± 74.8	147.2 ± 77.1
Lp(a), nmol/L, median (IQR)	93.0 (24 to 200), *n* = 227	51.5 (19 to 194)	45.0 (18 to 183), *n* = 1502	47.0 (19 to 185), *n* = 1475
PCSK9, µg/L	404.3 ± 111.3, *n* = 226	394.5 ± 174.6, *n* = 241	397.4 ± 152.9, *n* = 1501	390.8 ± 122.1, *n* = 1474

*ApoB*, apolipoprotein B; *ASCVD*, atherosclerotic cardiovascular disease; *BMI*, body mass index; *CHD*, coronary heart disease; *CeVD*, cerebrovascular disease; *CV*, cardiovascular; eGFR, estimated glomerular filtration rate; *HDL-C*, high-density lipoprotein cholesterol; *IQR*, interquartile range; *LDL-C*, low-density lipoprotein cholesterol; *Lp(a)*, lipoprotein(a); *PAD*, peripheral arterial disease; *PCSK9*, proprotein convertase subtilisin/kexin type 9; *PVD*, polyvascular disease; *SD*, standard deviation; *VLDL-C*, very-low-density lipoprotein cholesterol

### Efficacy

**Fig. 1:**
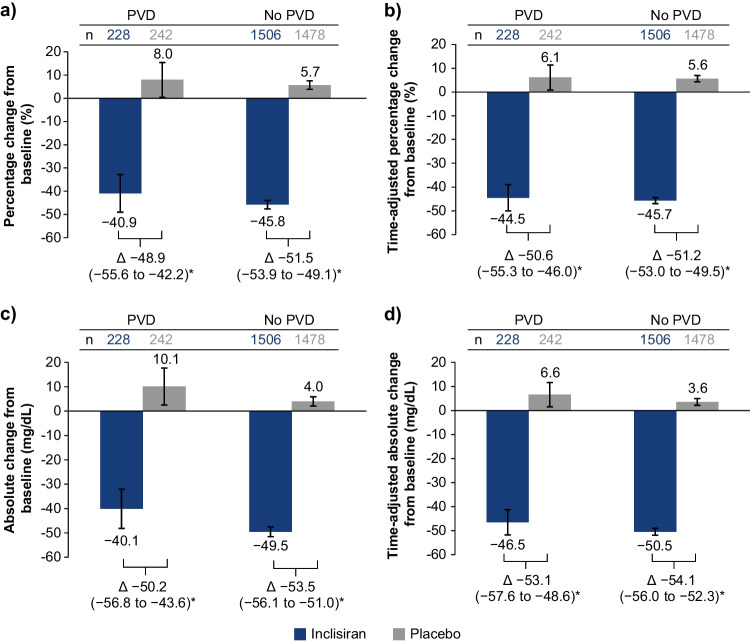
**Percentage** **and** **absolute** **changes** **in** **LDL-C**. **a** Percentage change from baseline to day 510, **b** time-adjusted percentage change from baseline after day 90 and up to day 540 in LDL-C, **c** absolute change from baseline to day 510, and **d** time-adjusted absolute change from baseline after day 90 and up to day 540. Data are presented as LS mean (95% CI); *p < 0.0001. CI, confidence interval; LDL-C, low-density lipoprotein cholesterol; LS, least squares; *n*, number of patients; PVD, polyvascular disease

The mean percentage change in LDL-C from baseline to day 510 and the mean time-adjusted percentage change from baseline after day 90 and up to day 540 were significantly greater in the inclisiran arm versus placebo, regardless of patients’ PVD status (Fig. 1A, B). The placebo-corrected percentage change in LDL-C from baseline to day 510 was −48.9% (95% confidence interval [CI], −55.6 to −42.2; *p* < 0.0001) for patients with PVD and −51.5% (95% CI, −53.9 to −49.1; *p* < 0.0001) for those without. The overall efficacy of inclisiran was consistent for patients with and without PVD (between-cohort interaction *p* = 0.80). The time-adjusted, placebo-corrected percentage change in LDL-C from baseline after day 90 and up to day 540 was −50.6% (95% CI, −55.3 to −46.0; *p* < 0.0001) and −51.2% (95% CI, −53.0 to −49.5; *p* < 0.0001) for patients with and without PVD, respectively. Similarly, the corresponding absolute and time-adjusted absolute changes in LDL-C from baseline were significantly greater with inclisiran versus placebo in each PVD cohort (Fig. 1C, D). For patients with and without PVD, the placebo-corrected absolute changes in LDL-C from baseline to day 510 were − 50.2 mg/dL (95% CI, −56.8 to −43.6; *p* < 0.0001) and −53.5 mg/dL (95% CI, −56.1 to −51.0; *p* < 0.0001), respectively, and the time-adjusted, placebo-corrected changes were − 53.1 mg/dL (95% CI, − 57.6 to − 48.6; *p* < 0.0001) and −54.1 mg/dL (95% CI, −56.0 to −52.3; *p* < 0.0001), respectively.

**Fig. 2:**
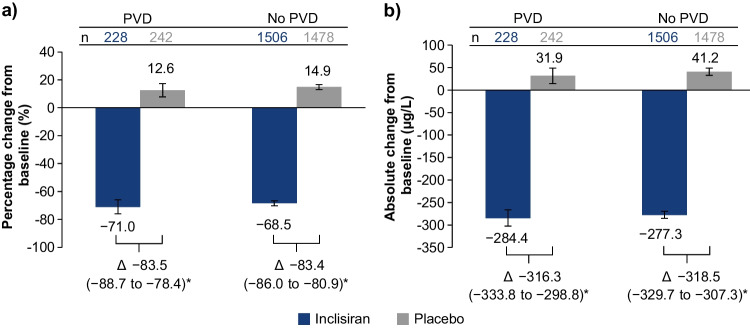
**Percentage** **and** **absolute** **changes** **in** **PCSK9** **at day** **510**. **a** Percentage and **b** absolute change from baseline to day 510 in PCSK9. Data are presented as LS mean (95% CI); *p < 0.0001. CI, confidence interval; LS, least squares; PCSK9, proprotein convertase subtilisin/kexin type 9; PVD, polyvascular disease

The mean placebo-corrected percentage and absolute changes in PCSK9 from baseline to day 510 are presented in Fig. 2, and percentage changes in other atherogenic lipids and lipoproteins [total cholesterol, non-HDL-C, VLDL-C, apoB, triglycerides, and Lp(a)] from baseline to day 510 are shown in Table 2. Overall, treatment with inclisiran significantly lowered PCSK9, total cholesterol, non-HDL-C, apoB, VLDL-C, triglyceride, and Lp(a) levels, regardless of patients’ PVD status. In particular, the placebo-corrected percentage change in apoB from baseline to day 510 was −42.6% (95% CI, −47.3 to −38.0; *p* < 0.0001) for patients with PVD and −42.1% (95% CI, −43.7 to −40.5; *p* < 0.0001) for patients without. The placebo-corrected percentage changes in non-HDL-C from baseline to day 510 were −47.8 (95% CI, −53.4 to −42.3; *p* < 0.0001) and −46.6 (95% CI, −48.5 to −44.6; *p* < 0.0001) for patients with and without PVD, respectively.

**Table 2:** Percentage change in other atherogenic lipids from baseline to day 510

Parameter	PVD			No PVD		
	Inclisiran (*n* = 228)	Placebo (*n* = 242)	Between-treatment difference	Inclisiran (*n* = 1506)	Placebo (*n* = 1478)	Between-treatment difference
Total cholesterol	−28.8 (−32.9, −24.7)	3.8 (−0.1, 7.7)	−32.6 (−36.8, −28.5)*	−29.8 (−30.8, −28.7)	2.9 (1.9, 4.0)	−32.7 (−34.1, −31.3)*
Non-HDL-C	−42.3 (−47.7, −36.8)	5.6 (0.3, 10.8)	−47.8 (−53.4, −42.3)*	−43.1 (−44.6, −41.7)	3.5 (2.1, 4.9)	−46.6 (−48.5, −44.6)*
ApoB	−39.7 (−44.4, −34.9)	3.0 (−1.6, 7.5)	−42.6 (−47.3, −38.0)*	−40.5 (−41.6, −39.3)	1.7 (0.5, 2.8)	−42.1 (−43.7, −40.5)*
VLDL-C^†^	−15.3 (−21.9, −8.6)	1.2 (−5.2, 7.6)	−16.5 (−23.2, −9.7)*	−6.3 (−8.2, −4.4)	2.6 (0.7, 4.6)	−8.9 (−11.5, −6.3)*
Triglycerides	−14.6 (−22.2, −7.0)	−2.7 (−4.6, −10.0)	−17.3 (−25.4, −9.2)*	−5.3 (−7.5, −3.0)	3.3 (1.1, 5.6)	−8.6 (−11.7, −5.5)*
Lp(a)^‡^, median (95% CI)	−16.8 (−21.9, −11.6)	4.8 (0.2, 9.5)	−21.6 (−28.3, −14.8)*	−16.1 (−17.8, −14.3)	4.3 (2.7, 5.9)	−20.4 (−22.2, −18.6)*

Data are presented as LS mean (95% CI) unless otherwise indicated

^*^p < 0.0001

^†^Calculated

^‡^Change in Lp(a) from baseline to day 540

*ApoB*, apolipoprotein B; *CI*, confidence interval; *HDL-C*, high-density lipoprotein cholesterol; *Lp(a)*, lipoprotein(a); *LS*, least squares; *PVD*, polyvascular disease; *VLDL-C*, very-low-density lipoprotein cholesterol

**Fig. 3:**
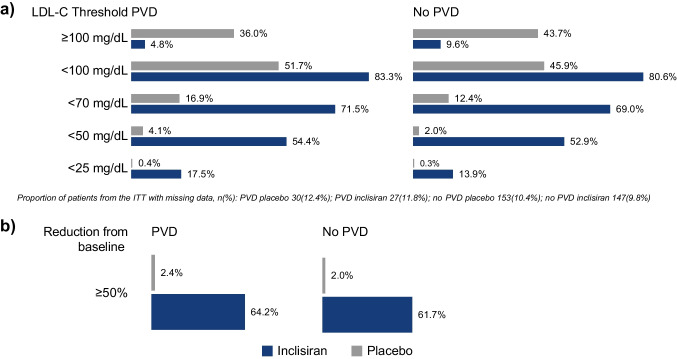
The proportion of patients achieving pre-specified LDL-C thresholds and a percentage reduction in LDL-C ≥ 50% at day 510. **a** The proportion of patients achieving pre-specified LDL-C thresholds at day 510 and **b** the proportion achieving ≥50% reduction in LDL-C levels from baseline at day 510. ITT, intention-to-treat; LDL-C, low-density lipoprotein cholesterol; PVD, polyvascular disease

For those with and without PVD, more inclisiran-treated patients achieved pre-specified LDL-C thresholds of <100, <70, <50, and <25 mg/dL at day 510 (Fig. 3A). In the PVD cohort, 83.3%, 71.5%, 54.4%, and 17.5% of inclisiran-treated patients achieved LDL-C levels of <100, <70, <50, and <25 mg/dL, respectively, on day 510. Similarly, among patients without PVD, 80.6%, 69.0%, 52.9%, and 13.9% of inclisiran-treated patients achieved pre-specified LDL-C levels of <100, <70, <50, and <25 mg/dL, respectively, on day 510.

The proportion of patients with a ≥ 50% reduction in LDL-C levels at day 510 was higher in the inclisiran arm for both cohorts versus placebo (Fig. 3B). Specifically, 64.2% of patients with and 61.7% without PVD who were treated with inclisiran achieved ≥ 50% reduction in LDL-C levels at day 510. Similarly, more inclisiran-treated patients in both the PVD and No PVD cohorts achieved pre-specified LDL-C thresholds and ≥ 50% reduction in LDL-C levels at any post-baseline visit (Supplementary Fig. 2).

### Safety

The safety population comprised 3449 patients, of whom 470 had PVD and 2979 did not. In the PVD cohort, 229 patients were in the inclisiran arm and 241 the placebo; in the cohort without PVD, 1505 and 1474 patients were in the inclisiran and placebo arms, respectively. The key safety findings are summarized in Table 3. Proportions of patients with reported TEAEs were largely similar between the treatment arms, regardless of PVD status. Clinically relevant TEAEs at the injection site, of which all were mild or moderate (none were severe), were reported more frequently with inclisiran versus placebo for both cohorts. TEAEs and TESAEs were reported more frequently in patients with PVD than in those without. The most frequently reported TEAEs are listed in Supplementary Table 2 and were largely similar between the treatment arms, regardless of PVD status. In the PVD cohort, more cases with bronchitis were observed in the inclisiran group compared with placebo (6.6% vs 2.1%; risk ratio [95% CI], 3.16 [1.17 to 8.55]). Proportions of patients with clinically relevant laboratory measurements were low and similar between treatment arms across cohorts. A greater number of patients with PVD had more than one clinically relevant laboratory measurement reported versus those without.

**Table 3:** Safety summary

Parameter	PVD		No PVD	
	Inclisiran (*n* = 229)	Placebo (*n* = 241)	Inclisiran (*n* = 1505)	Placebo (*n* = 1474)
Patients with ≥ 1 TEAE	186 (81.2)	202 (83.8)	1152 (76.5)	1117 (75.8)
Patients with ≥ 1 TESAE	70 (30.6)	93 (38.6)	283 (18.8)	313 (21.2)
Patients with ≥ 1 TEAE leading to study discontinuation	2 (0.9)	0 (0.0)	10 (0.7)	5 (0.3)
Clinically relevant TEAEs at the injection site				
Patients with ≥ 1 clinically relevant TEAE at the injection site	7 (3.1)	0 (0.0)	80 (5.3)	12 (0.8)
Mild	6 (2.6)	0 (0.0)	58 (3.9)	11 (0.7)
Moderate	1 (0.4)	0 (0.0)	22 (1.5)	1 (0.1)
Severe	0 (0.0)	0 (0.0)	0 (0.0)	0 (0.0)
Clinically relevant laboratory measurements				
Patients with ≥ 1 clinically relevant laboratory measurement	34 (14.8)	34 (14.1)	106 (7.0)	105 (7.1)
ALT > 3 × ULN	1 (0.4)	1 (0.4)	7 (0.5)	5 (0.3)
AST > 3 × ULN	1 (0.4)	3 (1.2)	7 (0.5)	6 (0.4)
ALP > 2 × ULN	2 (0.9)	0 (0.0)	5 (0.3)	5 (0.3)
Bilirubin > 3 × ULN	1 (0.4)	3 (1.2)	12 (0.8)	9 (0.6)
CK > 5 × ULN	3 (1.3)	2 (0.8)	18 (1.2)	19 (1.3)
Creatinine > 2 mg/dL	17 (7.4)	13 (5.4)	30 (2.0)	32 (2.2)
Platelet count ≤ 75 × 10^9^/L	0 (0.0)	1 (0.4)	1 (0.1)	1 (0.1)

Data are presented as *n* (%)

*ALP*, alkaline phosphatase; *ALT*, alanine aminotransferase; *AST*, aspartate aminotransferase; *CK*, creatine kinase; *TEAE*, treatment-emergent adverse event; *TESAE*, treatment-emergent serious adverse event; *PVD*, polyvascular disease; *ULN*, upper limit of normal

## Discussion

In this post hoc analysis of pooled data from three phase 3 trials, twice-yearly administration of inclisiran (after the initial and 3-month doses) in combination with a MTD of statins was shown to provide an effective and sustained lipid-lowering effect that was well tolerated in patients with PVD. These data suggest that the LDL-C-lowering effect and safety profile of inclisiran are preserved in patients with PVD. Previously, inclisiran has been shown effective and well tolerated in patients with HeFH and ASCVD and those at a high risk of ASCVD, achieving an ~50% decrease in LDL-C levels [25–27]. Among patients with ASCVD, those with PVD are at very high risk of CV death, MI, and ischemic stroke; thus, intensive LDL-C-lowering is critical in reducing this risk [8]. Notably, in the population presented here, the efficacy of inclisiran was similar in patients with PVD to those without (between-cohort interaction *p* = 0.80); by day 510, inclisiran treatment achieved placebo-corrected LDL-C reductions of 48.9% and 51.5% in patients with and without PVD, respectively. These findings are consistent with findings from a pooled analysis of all patients from the ORION-9, ORION-10, and ORION-11 trials [25], thus demonstrating the consistent effect of inclisiran across patient subgroups with increased risk.

Current guidelines for patients with a very high CV risk, defined as a history of multiple major ASCVD events (recent acute coronary syndrome, history of MI or ischemic stroke, symptomatic PAD) or one ASCVD event and multiple high-risk conditions, recommend lowering LDL-C levels to pre-specified goals to reduce the risk of CV events [12, 13]. However, a substantial proportion of patients do not achieve guideline-recommended goals with current LLT options [13, 19]. A key contributing factor to this is suboptimal long-term treatment adherence, where nearly half of patients discontinue treatment with high-intensity statins within 2 years [30]. Here, we show that with the addition of twice-yearly (after the initial and 3-month doses) dosing with inclisiran, ~71.5% of patients with PVD and 69.0% of those without achieved an LDL-C level of <70 mg/dL, and 54.4% and 52.9% of patients with and without PVD, respectively, achieved an LDL-C level of <50 mg/dL at day 510. Moreover, 64.2% and 61.7% of patients with and without PVD, respectively, achieved a ≥50% LDL-C reduction. It is important to note that for inclusion in the ORION-9, ORION-10, and ORION-11 trials, patients with ASCVD were required to have elevated LDL-C levels (≥70 mg/dL) despite a MTD of statins with or without additional LLT. Therefore, mean baseline LDL-C levels of patients within this analysis (>100 mg/dL and >110 mg/dL for patients with and without PVD, respectively) were higher than those typical for a general ASCVD population (~90 mg/dL) and other clinical studies investigating LLT in patients with PVD (86–96 mg/dL) [16, 29, 31]. This suggests that an even higher percentage of the general ASCVD population might achieve recommended LDL-C goals with inclisiran.

Treatment with inclisiran also significantly reduced levels of other atherogenic lipids and lipoproteins, including apoB by >42% and non-HDL-C by >46.4%, irrespective of PVD status. These results are of particular importance for patients with diabetes, obesity, metabolic syndrome, and hypertriglyceridemia, for whom the latest guidelines have defined secondary goals for non-HDL-C and apoB levels for more accurate risk assessments, even after the recommended LDL-C goal is attained [13].

This analysis demonstrates the well-tolerated safety of inclisiran in patients with and without PVD, where reported TEAEs, TESAEs, and clinically relevant laboratory measurements were similar across treatment arms, except for an excess of mild-to-moderate TEAEs at the injection site with inclisiran in both cohorts and a modest excess of bronchitis with inclisiran in patients with PVD. These findings are consistent with those from the overall ORION-9, ORION-10, and ORION-11 population, in which the majority of reported bronchitis events (all of which were self-limited) were mild-to-moderate [25]. The higher proportion of reported TEAEs in the PVD versus No PVD cohort was not surprising, considering the severity of PVD and the increased number of comorbidities in this population seen here and in other studies [16, 32]. Notably, similar findings were reported with other LLTs, with reported adverse events increasing proportionally with the number of vascular beds with atherosclerotic plaques [16]. While the data presented here demonstrate a promising safety profile, data from the ongoing long-term extension study (ORION-8 [NCT03814187]) and ongoing CV outcome trials (VICTORION-2 Prevent [NCT05030428] and ORION-4 [NCT03705234]) will be useful in confirming these observations of the effect of inclisiran over an extended period of time.

A limitation of the current analysis that warrants consideration is the relatively small sample size of the PVD cohort, which comprised only 470 patients after pooling data from the three phase 3 trials. Furthermore, patients were not screened for PVD during enrollment; PVD status was based on the information available from CRFs. Finally, while the benefit of using LLT to lower LDL-C levels and reduce CV risk has been widely established [18], the effect of inclisiran on CV events is currently being investigated in the ongoing CV outcome trials. Additional information on the long-term effect of inclisiran would be of particular interest, considering that the benefit of LDL-C reduction is compounded over time with long-term treatment [33].

## Conclusions

In conclusion, twice-yearly dosing with inclisiran (after the initial and 3-month doses), in combination with a MTD of statins with or without other LLT, consistently provides effective and sustained LDL-C lowering, along with reductions in levels of other atherogenic lipids and lipoproteins, in patients regardless of PVD status. Inclisiran was generally well tolerated in this subpopulation, except for a modest excess of mainly mild clinically relevant TEAEs at the injection site, which has been reported previously in the overall pooled population from ORION-9, ORION-10, and ORION-11 [25]. Considering that patients with PVD have a very high CV risk, infrequent dosing of inclisiran represents a potentially valuable therapeutic option for lowering LDL-C.

### Supplementary Information

Below is the link to the electronic supplementary material.

Supplementary file1 (DOCX 347 KB)

## Data Availability

Novartis is committed to sharing with qualified external researchers, access to patient-level data, and supporting clinical documents from eligible studies. These requests are reviewed and approved by an independent review panel on the basis of scientific merit. All data provided is anonymized to respect the privacy of patients who have participated in the trial in line with applicable laws and regulations. This trial data availability is according to the criteria and process described on www.clinicalstudydatarequest.com.
