# Supplementary material for: Efficacy and Safety of Inclisiran in Patients with Polyvascular Disease: Pooled, Post Hoc Analysis of the ORION-9, ORION-10, and ORION-11 Phase 3 Randomized Controlled Trials
Source: Cardiovasc Drugs Ther. 2022 Dec 23;38(3):493–503. doi: 10.1007/s10557-022-07413-0 (PMC11101568; doi:10.1007/s10557-022-07413-0)
Supplement: Supplementary file 1 — Supplementary file1 (DOCX 347 KB) [file 10557_2022_7413_MOESM1_ESM.docx]

**Supplementary Appendix**

This appendix has been provided by the authors to give readers additional information about their work.

Supplement to: **Efficacy and Safety of Inclisiran in Patients with Polyvascular Disease: Pooled, Post Hoc Analysis of the ORION-9, ORION-10, and ORION-11 Phase 3 Randomized Controlled Trials**

Koenig W, et al.

.

**Supplementary figures and tables**


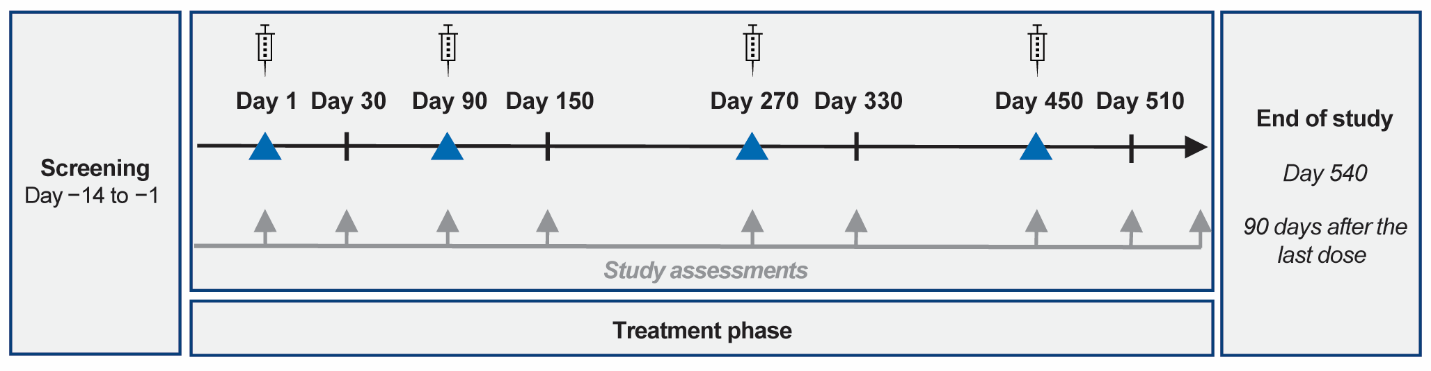


**Supplementary Figure 1. Study design and assessment schedule.**
Participants were randomized 1:1 to either 284 mg of inclisiran (equivalent to 300 mg of inclisiran sodium) or placebo, in combination with maximally-tolerated statins and/or other LLTs. LLT, lipid-lowering therapy.

**
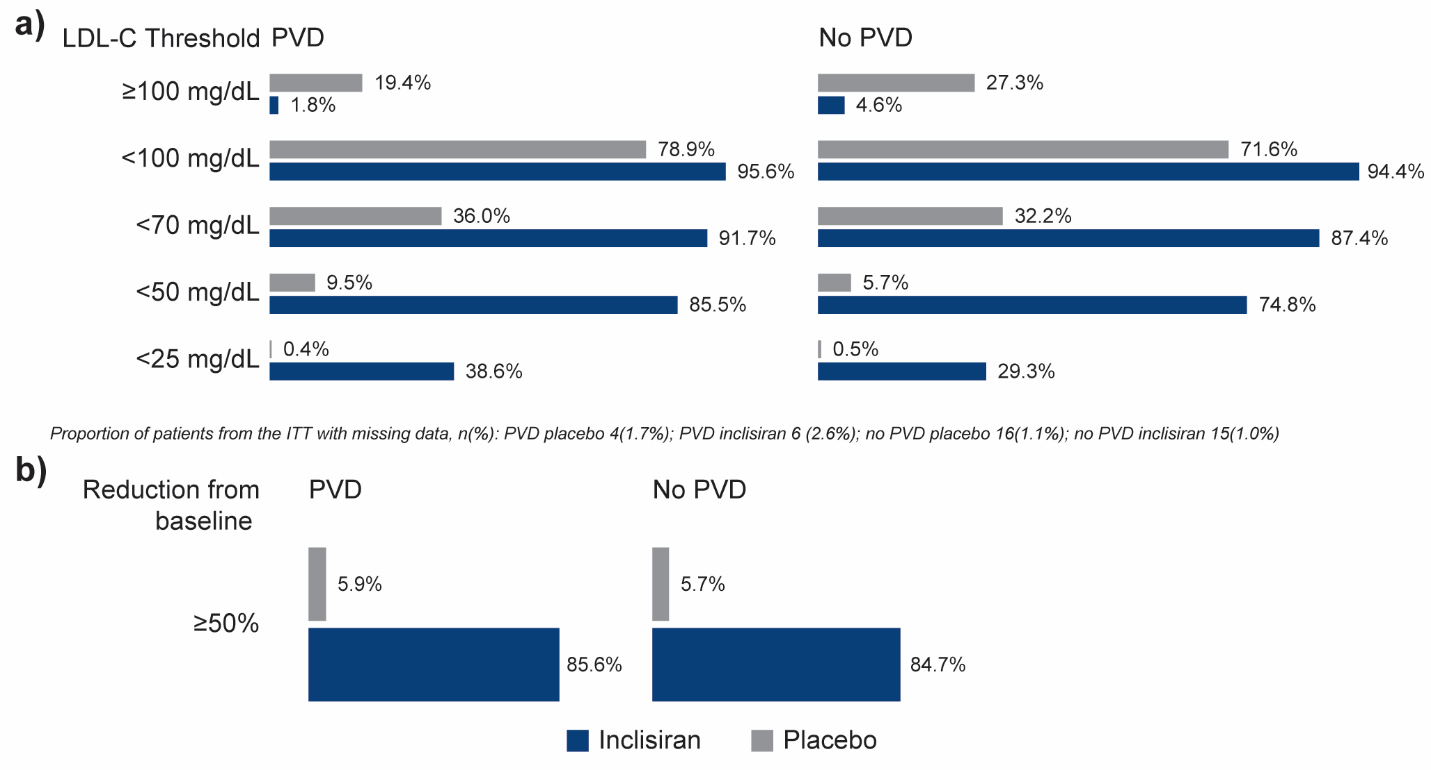
**

**Supplementary Figure 2. Proportion of patients achieving pre-specified LDL-C thresholds and a percentage reduction in LDL-C ≥ 50% at any post-baseline visit.**
(A) Proportion of patients achieving pre-specified LDL-C thresholds at any post-baseline visit (B) and the proportion achieving ≥50% reduction in LDL-C levels from baseline at any post-baseline visit. LDL-C, low-density lipoprotein cholesterol; PVD, polyvascular disease; ITT, intention-to-treat.

| **Supplementary Table 1.** Definitions of PAD, CAD and CeVD | |
| --- | --- |
| **Disease** | **Definition** |
| **PAD** | - Prior documented resting ankle-brachial index ≤0.85 - History of percutaneous or surgical revascularisation of an iliac, femoral, or popliteal artery - Prior non-traumatic amputation of a lower extremity due to PAD |
| **CAD** | - Prior MI - Prior coronary revascularisation (PCI or CABG) - Angiographic or CT-imaging evidence of coronary atherosclerosis (>70% stenosis in at least one major epicardial coronary artery) |
| **CeVD** | - Prior ischaemic stroke confimed by brain imaging (CT or MRI) and not caused by arterial fibrillation, valvular heart disease, or mural thrombus - Carotid artery stenosis >70% on prior angiography or ultrasound - Prior percutaneous or surgical carotid artery revascularisation |
| CABG, coronary artery bypass graft, CAD, coronary artery disease; CeVD, cerebrovascular disease; CHD, coronary heart disease; CT, computed tomography; CTA, computed tomographic angiography; MI, myocardial infarction; MRI, magnetic resonance imaging; PAD, peripheral artery disease; PCI, percutaneous coronary intervention. | |

| **Supplementary Table 2.** Summary of most frequent TEAEs (occurrence ≥5% in any treatment group) | | | | |
| --- | --- | --- | --- | --- |
| **Parameter** | **PVD** | | **No PVD** | |
|  | **Inclisiran**  **(n=229)** | **Placebo**  **(n=241)** | **Inclisiran**  **(n=1505)** | **Placebo**  **(n=1474)** |
| Diabetes mellitus* | 31 (13.5) | 31 (12.9) | 156 (10.4) | 149 (10.1) |
| Nasopharyngitis | 14 (6.1) | 18 (7.5) | 111 (7.4) | 100 (6.8) |
| Hypertension | 19 (8.3) | 20 (8.3) | 75 (5.0) | 76 (5.2) |
| Upper respiratory tract infection | 12 (5.2) | 9 (3.7) | 85 (5.6) | 83 (5.6) |
| Dyspnea | 15 (6.6) | 10 (4.1) | **-** | **-** |
| Back pain | 12 (5.2) | 13 (5.4) | **-** | **-** |
| Angina pectoris | 12 (5.2) | 12 (5.0) | **-** | **-** |
| Bronchitis^†^ | 15 (6.6) | 5 (2.1) | **-** | **-** |
| Fall | 13 (5.7) | 7 (2.9) | **-** | **-** |
| Cardiac failure congestive | 5 (2.2) | 13 (5.4) | **-** | **-** |
| Data are presented as n (%). *Diabetes mellitus represents worsening of glycemic control as defined in the clinical protocol; ^†^the risk ratio (95% CI) for bronchitis was clinically significant [3.16 (1.17 to 8.55)].  CI, confidence interval; PVD, polyvascular disease; TEAE, treatment-emergent adverse events. | | | | |
